# The Alaska Possibilities

**Published:** 1883-01

**Authors:** 


### THE ALASKA POSSIBILITIES.

The pressure to endow Alaska with a Territorail form of government evidently comes none too soon, if the recent reports of the mineral wealth of that region are to be relied on. Our Collector of Customs at Sitka says that lines of mines as large as the whole Comstock lode have already been brought into prominence. Last season a small party of surface diggers made $250,000. The assertion is made that in Alaska the richest mineral region in the world will be developed. In addition to this are the valuable timber, seal and other fisheries and, in the southern part, agricultural resources of no mean qualities. If there should be such a thing as a rush to the alleged El Dorado, it would be well to have some laws in operation to give title to claims, decide disputes, and administer justice generally. It may turn out that the Seward purchase of 1867 will prove a most valuable investment after all.

It seems that the United States has been treating its acquisition from Russia rather shabbily. The Russians before the transfer supported something like a system of schools among the Alents, who be came a comparatively intelligent people. Under our rule, or lack of it, this system has fallen to decay, and now but one or two schools remain. An effort is being made to secure a fund from the Government to revive these institutions and prove to the natives that they have not fallen wholly into the hands of barbarians. A very common error exists in regard to the climate of Alaska. In some very considerable section it is not uncomfortably cold, even in winter. A Sitka the mean winter temperature is 32.5° Fahrenheit and the mean summer temperature 54.6°. In other parts there is a very wide range of temperature during the year, it being very warm in summer and very cold in winter. At Fort Yukon it is said that the thermometer frequently rises above 100° in summer, and falls sometimes as low as 70° below zero in winter. Enough of the region is habitable certainly to permit people to live there if there is much money to be made by it.

The general impression of the extent of Alaska falls far short of the reality. In No. 2 of the circular of information issued within the year by the Bureau of Education, the facts, taken from an address of Dr. Sheldon Jackson, who has been interested in the cause of the schools there, are given. From the extreme north to the extreme south is 1,400 miles, or "as far as from Maine to Florida," From its eastern boundary to the end of the Aleutian Islands it is 3,200 miles, or "as far as from Washington to California, while the Island of Attu, at the end of the Aleutian chain is as far west of

San Francisco as Maine is east; so that between the extreme eastern and western sections San Francisco is the great central city." The national capital, however, will never be removed to San Francisco for this reason, there is rather more of this territory which does not count than there is of it which does. Still there may be a chance to carve half a dozen ordinary territories out of the part which is worth something, and the governing process had better begin, under the circumstances, at once.—*Globe Democrat.*

